# Plasmonic-nanowire near-field beam analyzer

**DOI:** 10.1515/nanoph-2023-0937

**Published:** 2024-02-13

**Authors:** Jian Peng, Runlin Zhu, Zhaoqi Gu, Hongyu Zhang, Lin Dou, Yanna Ma, Fuxing Gu

**Affiliations:** Laboratory of Integrated Opto-Mechanics and Electronics, School of Optical-Electrical and Computer Engineering, University of Shanghai for Science and Technology, Shanghai 200093, China

**Keywords:** plasmonic nanowires, micro/nano-waveguides, near-field beam analysis, 3D scanning, vortex beams

## Abstract

Experimental near-field analysis of the output beams from the end faces of micro/nano-waveguide is very necessary, because important information such as spatial intensity distributions, mode orders, and divergence angles can be obtained, and are very important for investigating and designing nanophotonic devices. However, as far as we know, it has not been demonstrated yet. In this work, we experimentally demonstrate a plasmonic-nanowire near-field beam analyzer, utilizing a single Au nanowire (AuNW) as the probe to scan the spatial near-field distributions of emitted beams from micro/nano-waveguide end-faces. Our analyzer can resolve the trade-off between high measurement resolution and light collection efficiency in conventional beam analyzers by a reverse nanofocusing process, achieving a probe resolution of 190 nm (<*λ*/8) and a simulated collection efficiency of ∼47.4 % at *λ* = 1596 nm. These attractive advantages allow us to obtain three‐dimensional (3D) scanning in a large range from the plasmonic hotspot region to the far-field region, characterizing the 3D spatial distribution evolution from a metal nanowire output beam for the first time, with an *M* ^2^ factor lower than that of the ideal Gaussian beam (*M* ^2^ = 1). In addition, the analyzer also demonstrates simultaneous characterization of multimodes in irregular and large-sized nanoribbons, further verifying its ability to selectively explore complex multimodes that are difficult to be predicted by numerical simulations. Our results suggest that this plasmonic-nanowire beam analyzer may hold promise for diverse near-field applications for micro/nano-waveguides such as nanolasers and biosensing, and offer a new method for understanding nanophotonic structures.

## 1 Introduction

Recently, micro/nano-waveguides have emerged as promising elements for tightly confining light energy and low-loss light propagating, and have found widespread applications such as in lasers, sensors, photodetectors, and nonlinear optics [1–4]. The confinement of optical fields at sub- or deep subwavelength scales in nanophotonic structures has become a significant research area [5–8]. Especially, the optical near fields at the end-faces can provide an important perspective on the interaction between guided modes and micro/nano-waveguides with mode confinement below the diffraction limits, such as spatial intensity distributions, mode orders, divergence angles, and group velocities. These factors are very important for investigating and designing nanophotonic devices such as nanolasers [9–12], scanning detection and imaging [13–16], and interference meters [17]. Therefore, experimental near-field analysis of the output beams from the end faces of micro/nano-waveguide is very necessary, but as far as we know, it has not been reported yet.

Simulation analysis methods, such as finite difference time domain (FDTD) calculation, are commonly used to analyze the near-field distribution characteristics of micro/nano-waveguide beams [18–19]. However, these simulation methods rely heavily on simple ideal cross-sections of the waveguide geometry, such as cylindrical and rectangular; however, in reality, the cross-sections and the end faces of the waveguides will vary with irregular shapes. In addition, for thick micro/nano-waveguides, many higher-order modes supported in waveguides are finally output at the end faces of the waveguides, resulting in strange spots that are difficult to be analyzed through simulation [20]. Therefore, a direct and precise experimental method is needed to characterize the various characteristics of the output beams.

Beam quality analyzers are essential tools for experimental evaluating and analyzing the spatial distribution of target beams and can provide valuable analysis of the excitation and transmission of different modes of laser in the waveguides [21]. Currently, commonly used solutions for near-field profiling of fibers or waveguides, as well as other applications where a beam of 50 µm or smaller is analyzed, mainly include camera-based analysis solutions and probe-based analysis solutions. In the camera-based analysis solutions, the sample beam is imaged by the microscope objective onto the camera detector array [22,23]. Obviously, using this approach will result in a loss of near-field information, and the resolution of the beam analysis is limited by the Abbe diffraction limit and the camera pixel resolution [23]. The probe-based analysis solutions use a probe detection method, and one of the representative applications is near-field scanning optical microscopy (NSOM), in which the nanoprobe acts as a near-field detector [5], [24–26]. However, this method has a contradiction between high measurement resolution and high energy collection efficiency, which is limited by the medium refractive index when using the single optical dielectric probe structure [27]. There is a limitation in the analysis of weak target beams when using the metal film coated structure above to improve the detection resolution [28,29]. In addition, although the NSOM-based system can achieve high-precision detection of sample morphology, the measurement of the output beam at the sample end face is still limited, especially in analyzing the 3D spatial distribution evolution of its output beam propagation from the near-field region to the far-field region [25].

In this work, we demonstrated a plasmonic-nanowire near-field beam analyzer that used an AuNW as the coaxial probe to achieve near-field scanning the output beams from end faces of micro/nano-waveguides. Our analyzer system has both high measurement resolution (190 nm at *λ* = 1596 nm) and high collection capability (∼47.4 % at 25 nm). For the first time, 3D spatial distribution evolution of an AuNW was experimentally achieved and analyzed. Finally, this beam analyzer also was well developed for the complex mode analysis application and selectively exploring diverse polarization modes in micro/nano-waveguides.

## 2 Principle and device design

**Figure 1:**
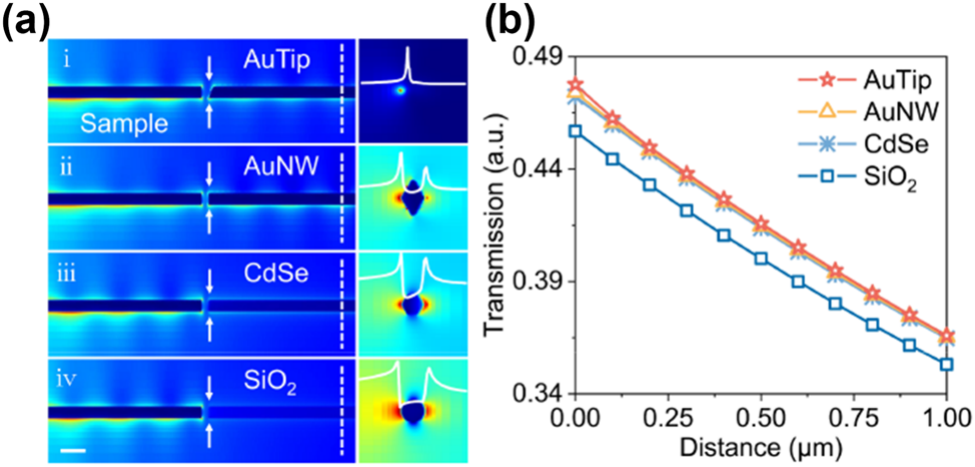
Principle for device design. (a) Numerical simulation of four air-coated probes with a diameter of 100 nm: a sharp-end AuNW (AuTip) and three uniform flat-ended Au, CdSe, and SiO_2_ nanowires, and their corresponding detection light fields at probe end-faces (scale bar, 200 nm). (b) Collection efficiencies of these four nanowire probes as a function of detection distance at the transmission distance on the probe of 1.5 μm.

We employed four types of nanowire probes to analyze their light collection performance. Figure 1(a) showed the morphology of these four air-coated probes with a diameter of 100 nm in the simulation, including one sharp-end AuNW (AuTip) and three uniform flat-end Au, CdSe, and SiO_2_ nanowires, which were used to detect a same AuNW sample. Simulation results show that when the propagation distance along these probes is 1.5 μm (dotted line in Figure 1(a)), the collection efficiency – defined as the fraction of energy carried by the sample end-face that is effectively converted into energy carried by the nanowire – of two AuNWs and one CdSe nanowire with the same sample-probe spacing range of 25 nm were approximately equal 47.4 %, while that of the SiO_2_ nanowire was slightly lower (∼45.4 %). The optical loss in this part is mainly the waveguide mode loss [14], and the transmission loss of the 1.5 μm nanowire probe only accounts for a small proportion. Furthermore, since the spatial resolution of the probe is primarily determined by its size, the plasmonic-AuNW probe with a sharp end could be manipulated to have better theoretical spatial resolution for near-field beam analysis. This improvement is attributed to its smaller mode area light collection resulting from the unique reverse nanofocusing process [14], [30–33]. Therefore, these structures are selected due to their ability of addressing the trade-off between high measurement resolution and efficient light collection found in traditional beam analyzers.

**Figure 2:**
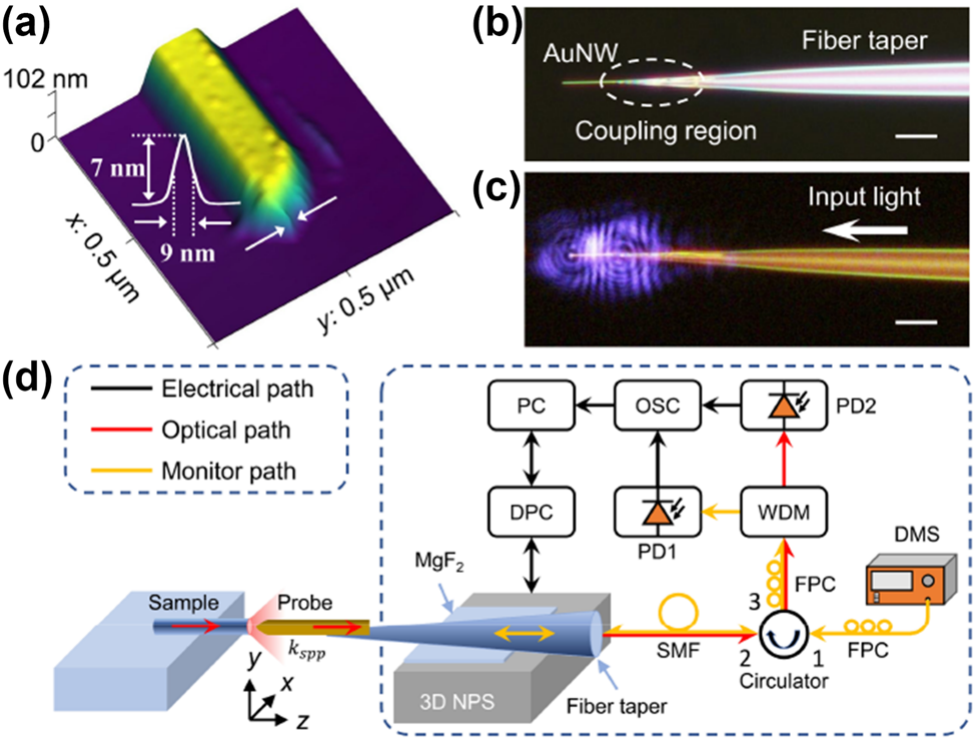
Experimental strategies for device design. (a) AFM image of an AuNW fabricated by a simple vapor transport method. (b) and (c) Plasmonic-nanowire probes that are constructed by an AuNW-fiber taper coupling structure and its guiding 1064 nm light (scale bar, 10 μm). (d) Experimental setup for the plasmonic-nanowire near-field scanning analyzer. 3D NPS, 3D nanopositioning stage. MgF_2_, magnesium fluoride. SMF, single-mode fiber. FPC, fiber polarization controller. DMS, distance monitoring source. WDM, wavelength division multiplexing. PD1 & PD2, photodetectors. OSC, oscilloscope. PC, personal computer. DPC, digital piezo controller.

Single-crystalline AuNWs were synthesized using a simple vapor transport method [34,35]. Figure 2(a) provides an atomic force microscopy (AFM) image of a fabricated typical AuNW, in which a one-dimensional nanostructure with uniform and smooth sidewalls can be seen and is conducive to low-loss light propagating. In addition, a sharp tip apex of the AuNW (<10 nm) can also be observed, which will produce a plasmon enhancement effect and provide better light field detection when used as a probe [14].

As it has been successfully demonstrated in polymer and semiconductor nanowires [36,37], using the suspension approach has the advantages of low-loss broadband waveguiding and high-efficiency coupling, which are also applicable to AuNWs for long-distance plasmon propagation [38–40]. As shown in Figure 2(b), an AuNW was supported by the tip of a suspended silica fiber taper. The nanowire was placed parallel to the fiber taper by micromanipulation, and the close contact had a few micrometers of overlap. Therefore, the optical near-field in the fiber taper and the nanowire will strongly overlap, resulting in efficient coupling of light from the fiber taper to the nanowire [8], [13–17], [23,38,40]. The close contact between the nanowire and the fiber taper can be maintained by strong van der Waals and electrostatic attraction [34,35], thus the coupling structure will remain stable throughout the experiments. Figure 2(c) shows the optical micrograph of a 150 nm-diameter AuNW supported by a fiber taper in air, and its guiding light at a wavelength of 1064 nm. A notable bright light spot was observed at the distal end of the nanowire with a surface plasmon polariton (SPP) propagation distance of about 13 μm from the taper fiber end, and no obvious scattering was observed during propagation. In addition, it has been calculated from the scattered spots that the coupling efficiency between the fiber taper and the AuNW is no less than 40 %. These low-loss waveguiding and high coupling efficiency characteristics makes it easy for AuNWs to collect the output light with low background noise, which is very important for sensing and detection applications.

The experimental setup for the plasmonic-nanowire near-field analyzer is shown in Figure 2(d). During the two-dimensional (2D) scanning process in the *x*–*y* plane, the plasmonic AuNW probe is located in the near-field region of the sample and captures the output light from the sample. Then, the captured light is transmitted along the nanowire in the form of a surface plasmon wave (*k*_spp_) [13–15], [38–40]. By changing the distance between the AuNW probe and the sample along the *z*-axis, beam spots at different positions can be obtained, thereby completing the analysis of the spatial beam distributions in 3D space (see Methods). The specific distance between the AuNW probe and the sample was calibrated using an “approach and retreat” method (see Figure S1). Unless otherwise specified, it was first set to be 25 nm.

## 3 Results and discussion

### 3.1 Calibration of the spatial resolution

**Figure 3:**
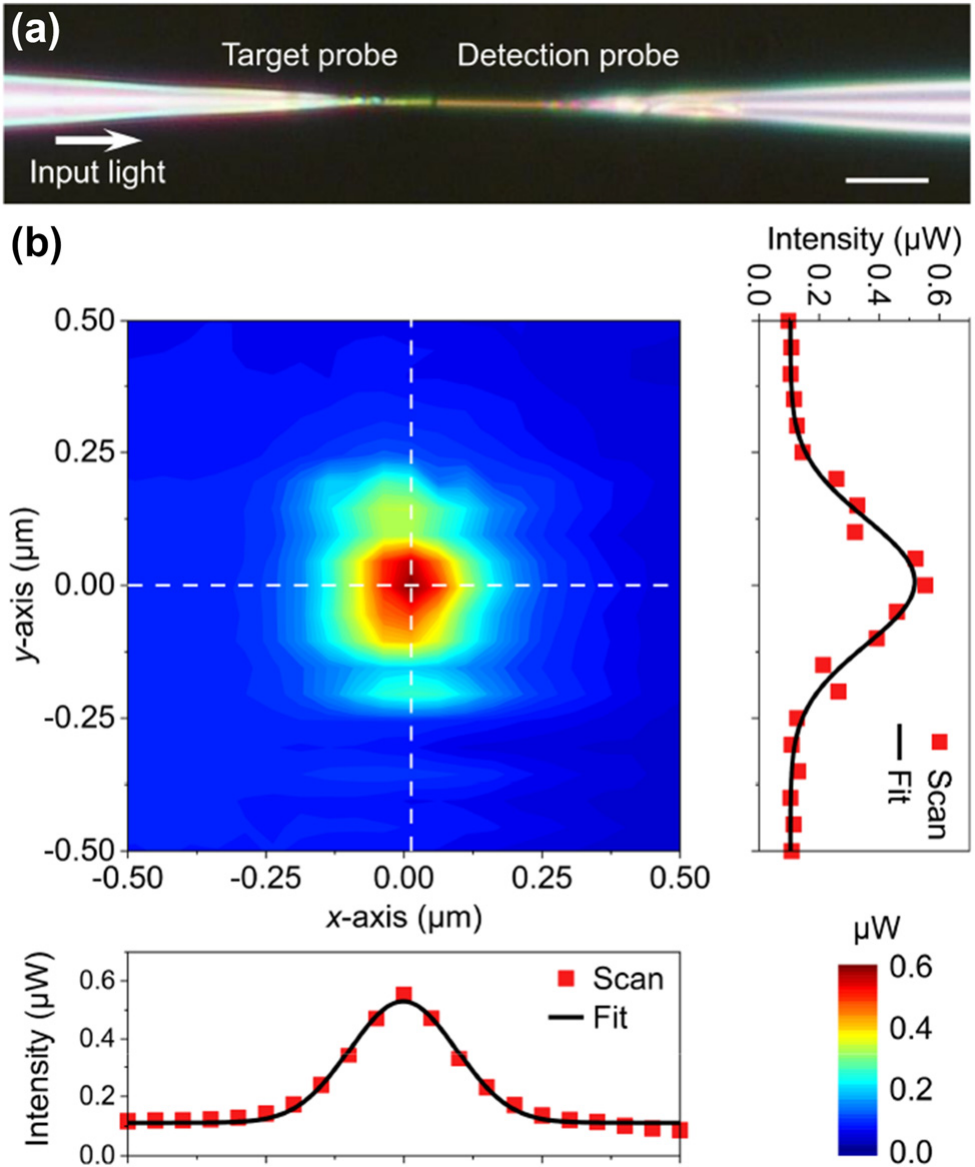
Calibration of the spatial resolution. (a) Microscope image of two similarly sized nanowire probes in a tip-to-tip structure (scale bar, 10 μm). (b) Transmitted intensity profiles at the minimum distance condition, with the cross-sections of the peak intensity profiles along the *x*-axis direction and the *y*-axis direction (red points), and their Gaussian fitting curve (black line).

As shown in Figure 3(a), two AuNW-fiber taper coupling units are used to set up a tip-to-tip structure for the calibration of the beam detection resolution. The detection light used was an L-band amplified spontaneous emission source (ASE) (1596 nm, 100 μW). The two AuNWs used had very similar dimensions, with a thickness of 130 nm and a height of 96 nm, as measured by the AFM. The left one serves as the target nanowire, and the right one serves as the detection nanowire, which can realize 3D light scanning and collection in the air. To achieve accurate beam detection, its scanning plane is set to be perpendicular to the wave vector of emitted beam from the target nanowire. Similar to the slit in a spectrometer or the hole in an aperture probe, the spatial resolution of the AuNW probe is primarily determined by its tip size, so AuNWs with smaller cross-sectional tip sizes are selected in this experiment. The beams emitted from different sample end faces are not always ideally symmetrical Gaussian shapes, so we need to define the beam radii (or beam half-width) of the sample beams on the *x*-axis and *y*-axis respectively, both of which are half the distance between two points on the light intensity distribution curve where the relative light intensity is 1/*e* ^2^ of the peak value.

The measured transmission intensity map is shown in Figure 3(b), in which the transmission intensity fields along the *x*-axis and *y*-axis are both well fitted by Gaussian distributions. The *x*-axis and *y*-axis in the figure represent the short-axis and long-axis directions of the detection beam, rather than the horizontal and vertical directions in the spatial distribution. The distortion of field distributions along the *y*-axis direction may be due to environmental vibration. Vibration in the *z*-axis will cause jitter in the gap between the probe and the sample, while vibration in the *x*-axis or *y*-axis will cause blurring of the measurement image, both of which will lead to a reduction in spatial resolution. Taking the measurement data along the *x*-axis as an example, the beam half-width of the transmitted beam is calculated to be 269 nm. Since the cross-sectional dimensions of the emitting and collecting nanowires are almost the same, the beam half-width of the AuNW-fiber taper coupling structure can be calculated as $1/\sqrt{2}$ times the measured profile beam half-width. That is, the corrected spatial resolution of the AuNW probe along the *x*-axis is 190 nm (see Supplementary Note 3), which is less than one-eighth of the light wavelength. The *y*-axis analysis follows the same approach, with a probe spatial resolution of 238 nm.

### 3.2 3D spatial distribution evolution of output beams

**Figure 4:**
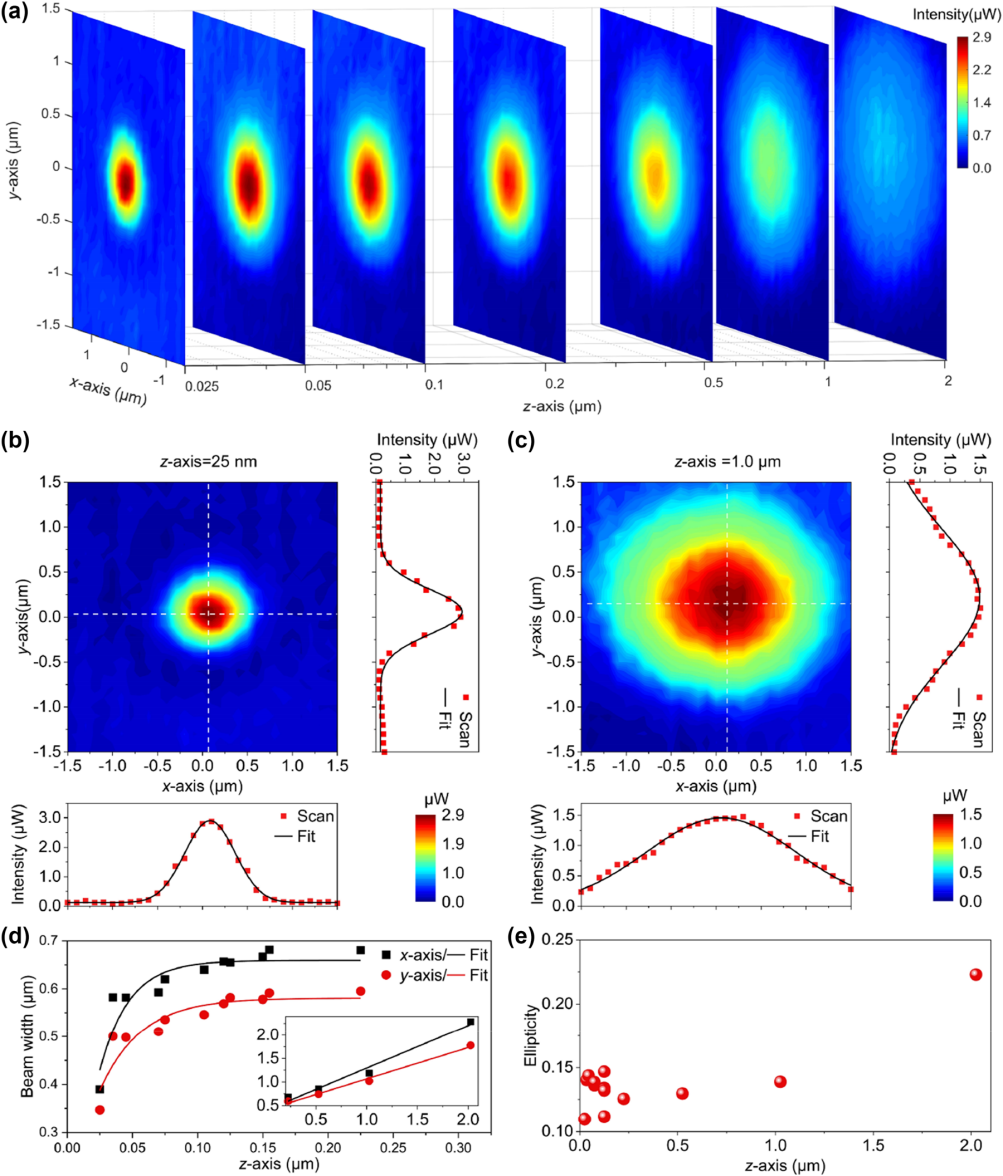
3D spatial distribution evolution of the AuNW output beam along (a) *z*-axis direction, and transmitted light field intensity distribution maps at the distances of (b) 25 nm and (c) 1 μm, respectively. The beam parameters change versus propagation distance: (d) beam radii along the *x*-axis (black square) and the *y*-axis (red circle), and (e) the ellipticity of the beam spatial profile.

To investigate the system’s 3D analysis capability, an AuNW with a diameter of 586 nm was selected as a new target sample. Its diameter is approximately five times the diameter of the right detection nanowire in Figure 3(a), which is similar to the size requirement of the aperture probe in the pinhole scanning method. Surface plasmons along the nanowire were also excited by the ASE source guided through a fiber taper. After 3D scanning, we obtained a series of typical transmitted light field intensity distribution maps at different distances, as shown in Figure 4(a). The optical signal is first transmitted in the AuNW in the form of surface plasmons, then emits at the end face and enters the air for further transmission. Remarkably, at the nanowire tip apex, the localized surface plasmons result in a highly confined electromagnetic field that is confined to a short distance from the tip apex and decays rapidly (see Figure S3), exhibiting a distance-dependent divergent evolution phenomenon. To further study the evolution characteristics of the beam spatial distribution, two transmitted light field intensity distribution maps at the distances of 25 nm and 1 μm are extracted, as shown in Figure 4(b) and (c), which are in the plasmonic hotspot region and the optical transmission region, respectively. The beam waist radii of the two-dimensional Gaussian fit in Figure 4(b) were calculated to be about 561 nm in the *x*-axis direction and 500 nm in the *y*-axis direction, with an ellipticity of 0.109. And those in Figure 4(c) were calculated to be about 1.187 μm in the *x*-axis direction and 1.022 μm in the *y*-axis direction, with an ellipticity of 0.139.

In the region of local enhancement of the SPP signal located near the nanowire tip apex (plasma hotspot region) [41], the beam widths decays rapidly with a near exponential divergence until reaching a distance of about 250 nm from the nanowire tip apex, as shown in Figure 4(d). As the distance continues to increases, the beam begins to diverge linearly with distance (inset of Figure 4(d)), as is usually measured in the far-field region. The slopes of the linear fits to the *x*-axis and *y*-axis beam radii in this region are 0.884 and 0.684, respectively, corresponding to the beam divergence angles of 41.5° and 34.4°, respectively. The difference of calculated values between *x*-axis and *y*-axis may be caused by the geometry of nanowires and the axis misalignment between the sample and probe nanowires during 3D scanning.

Similar to the beam width, the ellipticity of the sample beam spatial profile also undergoes an evolution from plasmon transmission to free-space transmission, which is shown in Figure 4(e) with a distance-dependent evolution characteristic. The beam profile at the AuNW end face is closely related to its size, and the beam transmitted in the far field shares similar transmission characteristics with the usual lens-focused beam, but its ellipticity gradually increases due to different beam divergence angles on the *x* and *y*-axis. The measurement errors of the beam width and ellipticity in the plasma hot spot area may be due to positioning errors of the nano-positioning stage and environment disturbance. Since the existence of the axis misalignment, the ellipticity of the measurement beam increases with the increasing measurement distance. However, the beam analysis system still has relatively accurate measurement capabilities within 1 μm distance, with an ellipticity error of 0.037. Beyond this range, the divergence distortion may occur.

The *M* ^2^ factor is also an important evaluation index for beam analysis. For an ideal Gaussian beam, the product of the beam width at the waist and the beam divergence angle is usually constant. When taking the end face of the AuNW as the beam waist for beam analysis, due to the existence of the plasmonic hotspot region, spatial distribution size of the beam is usually smaller than that of the lens-focused beam or the ordinary single-mode fiber beam, thus the *M* ^2^ factors of this AuNW were calculated to be 0.884 in the *x*-axis direction and 0.684 in the *y*-axis direction, which are lower than that of the ideal Gaussian beam (*M* ^2^ = 1). This is one of the distinguishing characteristics of plasmonic nanowire output beams that are far below the diffraction limit. Thus, our analyzer system can obtain sufficient beam analysis parameter values with high accuracy. To the best of our knowledge, this is the first time that the beam evolution of a micro/nano-beam from the near-field region to the far-field region has been experimentally detected.

### 3.3 Complex mode analysis for thick nano-waveguides

**Figure 5:**
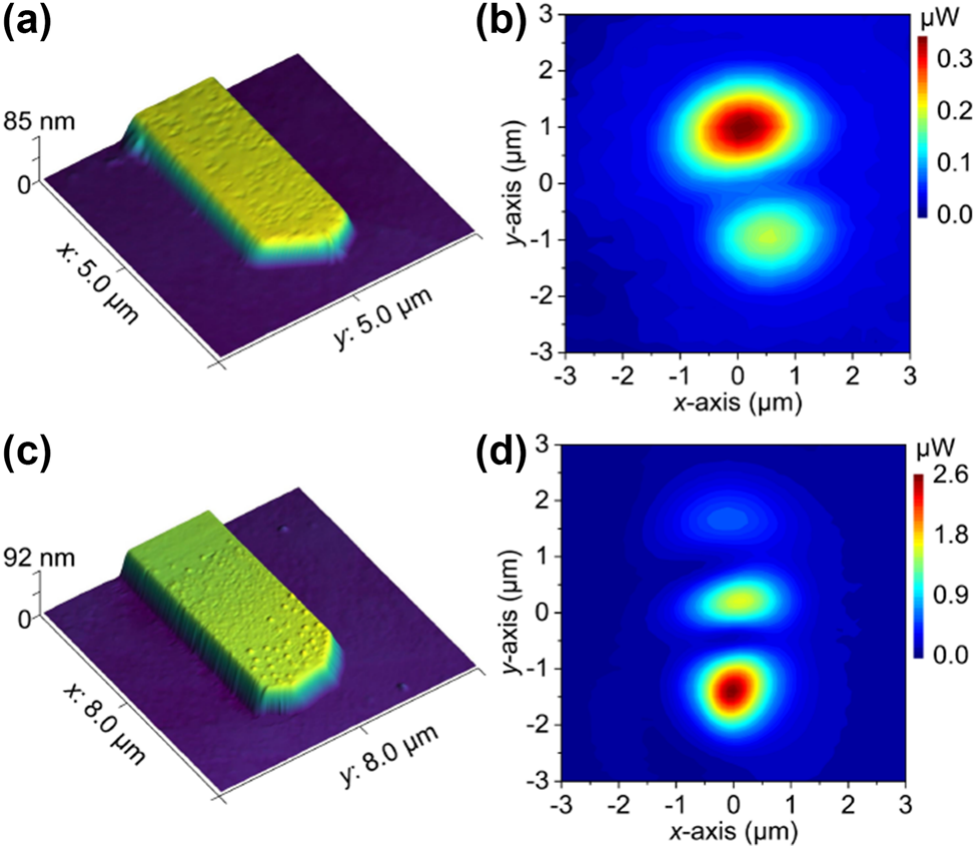
Beam field intensity distributions of two thick Au nanoribbons with irregular end-face types. AFM images of the two nanoribbons end faces are shown in (a) and (c), and the measured corresponding intensity distributions are shown in (b) and (d), respectively.

To expand our study and uncover complex mode fields at sub-wavelengths, two extra thick nanoribbons with irregular end-face dimensions were also investigated. The irregularities at the end faces are mainly caused by the crystal growth mechanism of the metal. One nanoribbon with a width of 1.59 μm and a height of 85 nm was shown in Figure 5(a), and its measured beam intensity distribution was shown in Figure 5(b), exhibiting a typical multimode behavior with a two-spot spatial distribution. Larger waveguide with a width of 2.90 μm and a height of 92 nm, as shown in Figure 5(c), demonstrated more spots in beam spatial distribution. As shown in Figure 5(d), the minimum beam half-width of the multi-peak intensity fields (center spot) is measured to be ∼455 nm. In thick nano-waveguides, co-transmission multiple modes with different transmission losses can be supported, and they will then emit from the waveguide with different wavevector directions. However, these emitting multimodes are difficult to be measured and simulated directly. Here, we can simultaneously obtain beam near-field spatial distribution information of all modes in different micro/nano-waveguides without additional processing. And this application can also be extended to the characterizations of more waveguides with complex topography or end-face features, which allows us to deeply study the impact of geometric topography on the near-field emission characteristics of small-scale waveguides, and then explore new optical modes.

### 3.4 Exploring diverse polarization modes

**Figure 6:**
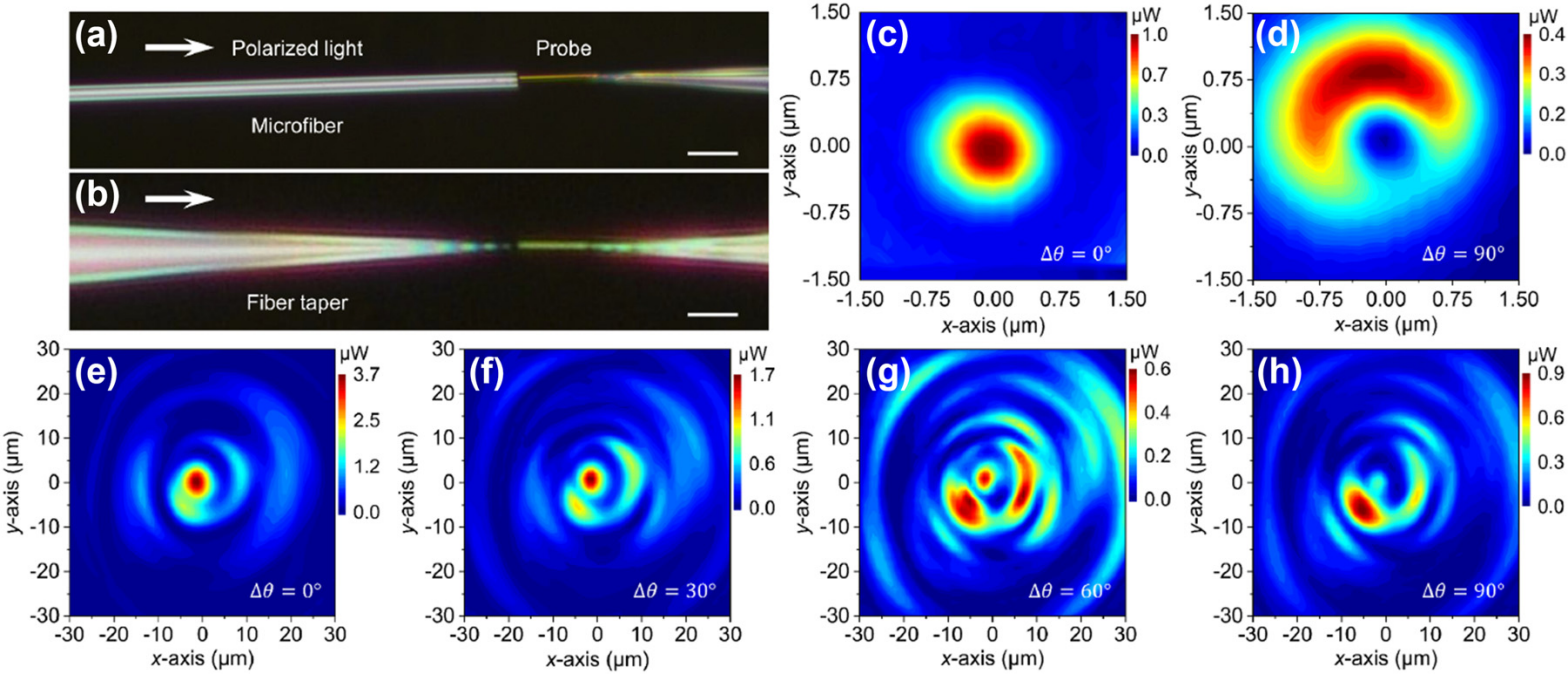
Exploring diverse polarization modes. Microscope images of (a) a microfiber and (b) a fiber taper characterized by the plasmonic-nanowire probe of the beam analyzer (scale bar, 10 μm). Transmitted mode field distributions of the microfiber at two polarization states (c) 0° and (d) 90°. (e)–(h) Transmitted mode field distributions of the fiber taper as the input polarization state changed from 0° to 90°.

Our beam analysis system also demonstrated its potential in exploiting diverse polarization modes. Here the source was replaced by a tunable linearly polarized light source (TSL-550, Santec), whose wavelength was set to 1596 nm and an FPC was used to adjust its polarization state. For comparison, the samples in Figure 6(a) and (b) were a microfiber with a uniform diameter and a fiber taper with a sharp tip (tip diameter usually less than ∼100 nm), respectively, both of which had multimode transmission, thus beams emitted from the end faces of the fibers often contain multiple modes [20].

Figure 6(c) and (d) showed the output beam distributions of the microfiber in two mutually perpendicular polarization states at the same transmission distance. Different patterns were observed, confirming that they were derived from the fundamental mode and higher-order modes, respectively. To elaborate in more details, Figure 6(e)–(h) demonstrated the mode evolutions of vortex-like beam output from a fiber taper, by gradually changing the polarization per 90 degrees of its input light. Initially, the output beam detected by the analyzer probe in Figure 6(e) has the highest intensity spot at the center position (mainly fundamental mode), with a beam width of 4.6 μm. As the polarization state is adjusted, the intensity of the central spot weakens, and a clear vortex-like light field distribution appears. The light field energy at large ring radii gradually increases (shown in Figure 6(f) and (g)), i.e., the light field energy in the higher-order modes gradually increases. When the polarization state of the incident light is rotated by *π*/2, the energy of the vortex-like beam returns to the area with a smaller ring radius and is asymmetrical, as shown in Figure 6(h), where both higher-order modes and the fundamental mode propagating in the waveguide experience greater attenuation. When continuing to rotate the polarization state of the incident light by another *π*/2, the output beam distribution returns to the initial state shown in Figure 6(e), suggesting a polarization adjusting period of *π*. Thus, by analyzing the polarization-dependent spatial distributions of the vortex-like beam output, we can explore various new modes that are difficult to be predicted by numerical simulations.

## 4 Conclusions

In summary, we have experimentally demonstrated a plasmonic-nanowire near-field beam analyzer, which uses a single AuNW as the probe to scan the 3D spatial distributions of the beams emitted from the end faces of micro/nano-waveguides. Our analyzer system can resolve the contradiction between the high measurement resolution and high light collection capability in conventional beam analyzers by a reverse process of nanofocusing. This approach effectively enhances, extracts, and guides emission from the nanoscale to a photonic fiber taper, realizing ultra-high resolution while keeping relatively low plasmonic losses. At *λ* = 1596 nm, a probe resolution of 190 nm (<*λ*/8) and a simulated collection efficiency of ∼47.4 % were obtained. These attractive advantages allow us to obtain 3D scanning in a large dynamic range from the plasmonic hotspot region to the far-field region, and thus we obtained the first-ever 3D spatial distribution evolution from the output beam of a metal nanowire. The *M* ^2^ factors were 0.884 in the *x*-axis direction and 0.684 in the *y*-axis direction, which are lower than that of the ideal Gaussian beam (*M* ^2^ = 1), showing a plasmonic nanowire output beam that is far below the diffraction limit. In addition, the analysis system also achieved simultaneous characterization of multi-mode beam transmission in irregular and large-sized nanoribbons, further verifying its ability to selectively explore complex transmission modes that are difficult to be predicted by numerical simulations. Our results suggest that the plasmonic-nanowire beam analyzer may have broad near-field applications for micro/nano-waveguides, such as plasmonic waveguides, biosensing, nanolasers, and other photonic devices.

## 5 Methods

### 5.1 Hybrid structure fabrication and distance calibration

As-fabricated AuNWs were first transferred in parallel by micromanipulation to the tip of the suspended silica optical fiber tapers on an MgF_2_ substrate. To efficiently excite the plasmon propagation in AuNWs, we used an evanescent-wave coupling technique [4], [16], [17], [37–40]. In our approach, excitation light was first coupled into a standard silica fiber (SMF-28, Corning) and then squeezed into a fiber taper with a tip diameter of less than 100 nm. The fiber tapers were drawn from the standard optical fiber by using a simple flame-heated method [34,36 39 40], and then were bound to an MgF_2_ substrate using a low-index UV-cured fluoropolymer. The tip of the fiber taper was protruded out of the substrate with a distance of 120 μm. The AuNWs were picked up from the growth substrates by a fiber taper with a sharp tip via micromanipulation and placed parallelly at the tip of the suspended silica fiber taper in air.

To calibrate the distance between the AuNW probe and the sample, an “approach and retreat” method was used (see Supplementary Note 1). A tunable narrow-linewidth light source (TSL-550, Santec) with a wavelength of 1550 nm and a power stability of ±0.05 dB/8 h was employed as the excitation light source. After a fiber polarization controller (FPC), a circulator (ports 1 to 2), and a fiber taper, the emitted light excited the plasmonic-nanowire probe. The reflection light from the sample was also collected using the same probe and circulator, and directed to the photodetector 1 (PD1) (OE-300-IN-03, FEMTO) through a wavelength-division multiplexer (WDM).

### 5.2 Near-field beam characteristics and analysis

To investigate the system’s ability to analyze beam quality, the AuNW-fiber taper-MgF_2_ structure was mounted on a triple-axis micromanipulator (P-611.3S, Physik Instrumente) for near-field beam scanning. It has a travel range of 100 μm, a system resolution of 1 nm (closed loop), and a unidirectional repeatability of ±10 nm in three dimensions. During the scanning, the distance calibration source was shut down. The AuNW positioned in the sample’s near-field region could capture the light field intensity distribution of the beam at different scanning positions. Signals collected by the AuNW probe were directed to photodetector 2 (PD2) using an optical circulator and a WDM and then analyzed using an oscilloscope (OSC) module (NI PXI-5152, National Instruments) and a computer.

## Supplementary Material

Supplementary Material Details
