# Supplementary material for: Plasmonic-nanowire near-field beam analyzer
Source: Nanophotonics. 2024 Feb 13;13(6):881–9. doi: 10.1515/nanoph-2023-0937 (PMC11501462; doi:10.1515/nanoph-2023-0937)
Supplement: Supplementary file 1 — Supplementary Material Details [file j_nanoph-2023-0937_suppl_001.pdf]

Supporting Information

Plasmonic-nanowire near-field beam analyzer

Jian Peng, Runlin Zhu, Zhaoqi Gu, Hongyu Zhang, Lin Dou, Yanna Ma*, and Fuxing Gu*

Laboratory of Integrated Opto-Mechanics and Electronics, School of Optical-Electrical and Computer Engineering, University of Shanghai for Science and Technology, Shanghai 200093, China;

*Corresponding Authors:
Yanna Ma, e-mail: mayanna@usst.edu.cn;
Fuxing Gu, e-mail: gufuxing@usst.edu.cn

Supplementary Note 1: Evaluation of absolute tip distance

Before performing beam analysis on each sample, we need to calibrate the distance between the probe and the sample using an operation named “approach and retreat”. The specific process is as follows:

First, the sample was placed on an MgF_2 substrate by micromanipulation, partially suspended outside the substrate, and oriented parallel to the AuNW probe. During the coaxial calibration process, the sample and substrate are kept in close contact through strong van der Waals forces and electrostatic attraction. The nanowire probe then slowly approaches the sample through a 3D nanopositioning stage (NPS) until a "soft contact" occurs, where the received light intensity suddenly weakens, and the AuNW probe immediately retreats. Typical experimental results of reflected light intensity versus sample-probe distance show that there is a clear jump point in the optical intensity when a contact event occurs (**Figure S1**). But when the NPS retreats, the change in light intensity is not immediately responsive and there is a hysteresis of about 25 nm, which may be caused by the attraction between the probe and the sample. Therefore, we can estimate the relative distance between the sample and the probe from the optical parameter change curve, which is generally about 25 nm. This calibration method proved critical to obtaining accurate measurements in our experiments of beam analysis, ensuring that our near-field measurements were reliable and consistent.

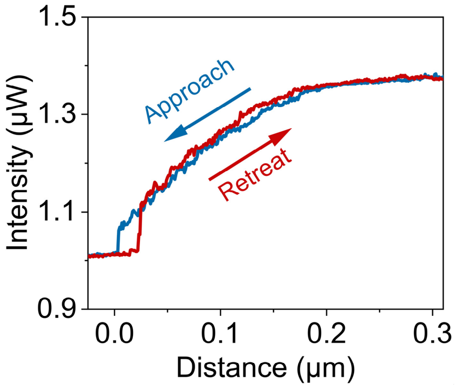

Figure S1. Variation of reflected optical intensity with distance.

Supplementary Note 2: Numerical simulations of plasmonic-nanowire radiation in near-field and far-field regions

To evaluate the beam emission characteristics of the AuNW samples, we used finite difference time domain (FDTD) software to analyze the coupling of metal nanowires and light-guiding fiber taper and light field distribution in the near field of the nanowires. This will guide subsequent experimental work.

The taper angle of the fiber taper (dashed outline in **Figure S2**(a)) is defined as 6°. The cross-section of the AuNW is a prism with a side length of 100 nm, a coupling length of 5 μm, and a suspended length of 5 μm, and the end face of the nanowire is arranged at an angle of 45°. The wavelength of the input light is set to 1596 nm and propagates in a vertically polarized quasi-TE mode, which can be perfectly coupled to the nanowire and propagates efficiently.

Optical coupling of the AuNW and light-guiding fiber taper:

We first simulated and analyzed the transmission characteristics of the fiber taper to the AuNW end face, as shown in **Figure S2**(b), with a transmission efficiency of 8.9%. Comparing with the transmission efficiency of the single fiber taper probe (15.1%), the input optical signal can be effectively coupled into the nanowire through the fiber taper, and then the low-loss surface plasmon wave propagates along the nanowire to the end face and generates localized plasmon enhancement effect at the tip apex, realizing a classic quasi-adiabatic nanofocusing process.

Figure S2(c) shows the beam spatial distribution of the 2D light field at a distance of 25 nm from the end face of the AuNW, where the output beam has evolved into an approximately Gaussian light spot. When the distance between the monitor (dashed line in **Figure S2**(c)) and the AuNW probe end face increases, the beam spatial distribution acquired at different distances

along the x-axis direction is shown in **Figure S2**(d). It can be seen that as the distance increases, the spot of the output beam gradually diverges, that is, the measured beam width increases rapidly, while its light field intensity decreases rapidly. The data of the beam radius acquired at different distances during the measurement process are extracted in **Figure S2**(e), which further demonstrates the compression characteristics of beam radius as the distance decreases.

Similarly, due to the reciprocity of the optical path, when the AuNW-fiber taper coupling structure is used as a detection probe, higher transmission efficiency can also achieve efficient coupling and signal transmission between the AuNW and the fiber taper. After the beam information is collected by the AuNW, and coupled to the fiber taper, it can be transmitted to the far field through the optical fiber for signal collection and processing. Since the simulation results show that the resolution of the AuNW probe is dominated by the distance between the probe and the sample, we use the "approach and retreat" approach to control the distance between the two at ~25 nm, thereby achieving a theoretical detection resolution of 94 nm.

It is worth noting that this beam analysis system is not limited to analyzing the beam emitted by the sample guided by the microfibers. For luminescent samples excited by the spatial light, their beams can also be captured and analyzed accurately.

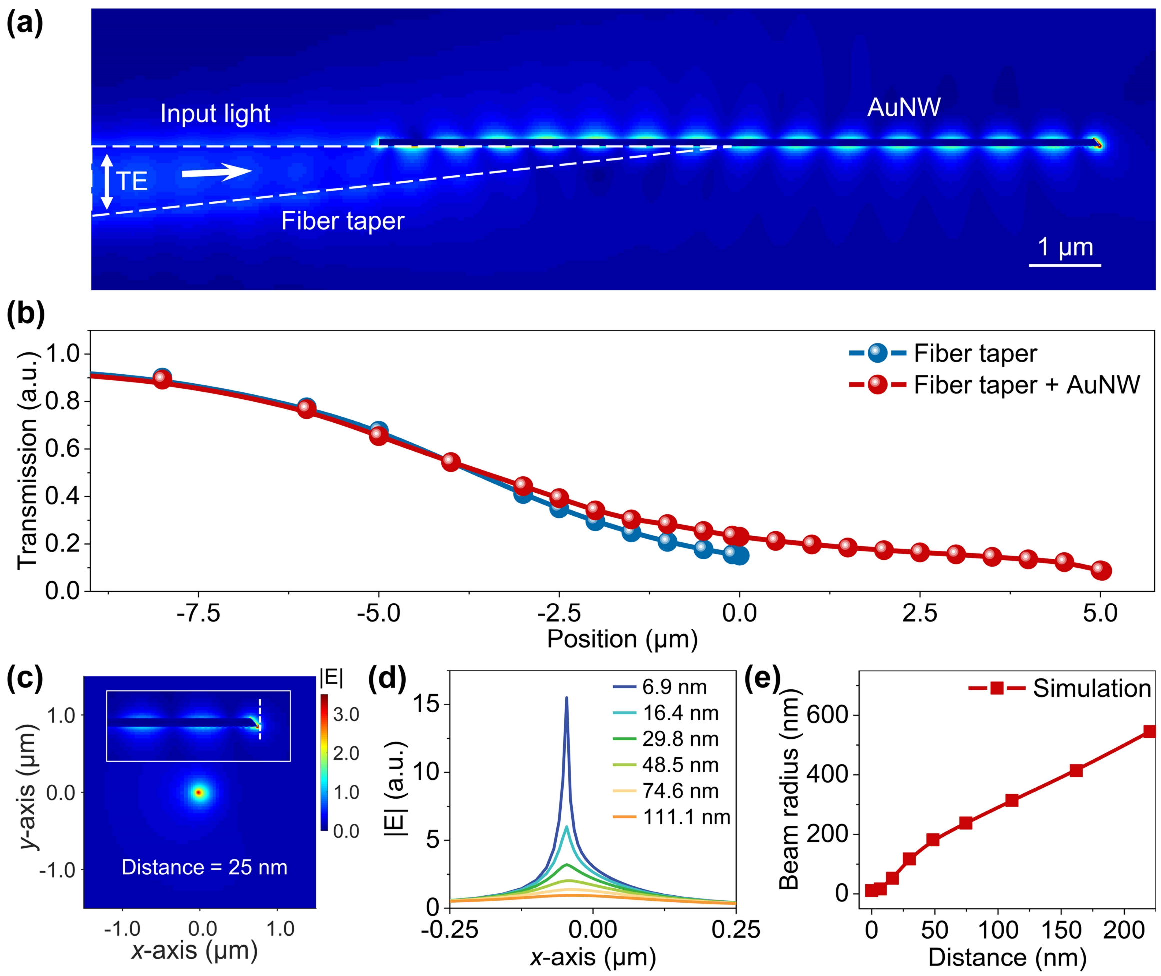

Figure S2. (a) Numerical simulations of the electric field distribution of a AuNW-fiber taper probe in the quasi-TE mode with vertical polarization. (b) Transmission charactersistics of a single fiber taper probe and a AuNW-fiber taper probe. (c) Electric field distribution in the cross-section at a distance of 25 nm from the nanowire tip. The inset shows the result of the electric field radiation from the nanowire tip. (d) Electric field distribution curves along the *x*-axis at different distances from the spot center cross-section, and (e) the corresponding beam radius as a function of distance.

In the near-field region of the AuNW tip:

The behavior of electrons at the metal-dielectric interface results in the excitation of localized surface plasmons, resulting in a highly confined electromagnetic field near the nanowire tip, and then exits at the end face of the waveguide and enters the air for further transmission.

Due to the growth characteristics of the crystal, AuNWs usually obtain sharp end faces, and their output beams exhibit transmission characteristics related to their metallic properties

in the near-field region. Optical radiation from the plasmon hotspot region in the AuNW near field is related to the unique properties of the electron cloud on the metal surface, which can support the generation of surface plasmon resonance (SPR) at the sample surface, resulting in a highly confined electromagnetic field near the nanowire tip apex, as shown in **Figure S3** [1-3]. As can be seen, the electromagnetic field is limited to a very short distance from the tip. As the transmission distance increases, the energy of the electromagnetic field decreases rapidly, and the energy attenuation reaches 10 dB at a distance of 250 nm. Outside the plasmon hotspot region, the electromagnetic field emitted from the waveguide retains its wave-like properties, but exhibits an evolution of the spatial distribution on the subwavelength scale, leaving only larger size information for transmission to the far field.

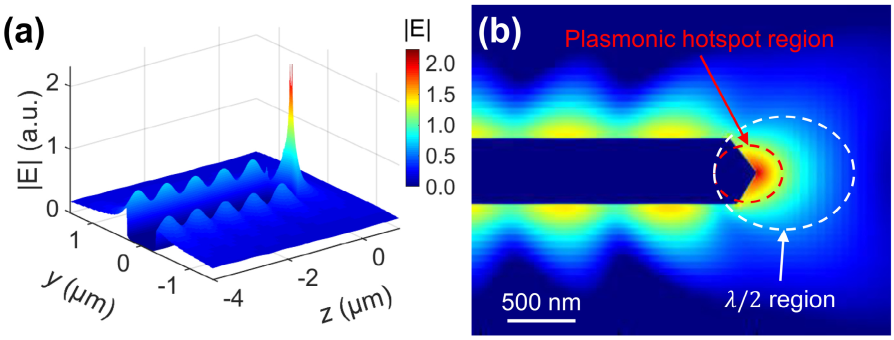

Figure S3. Near-field numerical simulation of the output beam from an AuNW tip. (a) 3D and (b) 2D (logarithmic color scale) electric field distributions of the output beam.

Supplementary Note 3: Experimental Calibration of Resolution

To experimentally achieve single-probe resolution calibration, we first utilize the tip-to-tip configuration to assess the transmission properties of the nanowire probe as a near-field beam analyzer. Subsequently, we employ the deconvolution of two Gaussian functions to complete the characterization of the optical field width of a single nanowire. The transmitted intensity distribution resulting from the spatial transmission of two single-mode optical fields can be approximated as an elliptical Gaussian distribution, meaning that the intensity can be expressed as

$$\mathrm{I}(x,y)=I_0 e^{-2\left(\frac{x^2}{w_x^2}+\frac{y^2}{w_y^2}\right)}, \tag{1}$$

where I_0 represents the peak intensity. w_x and w_y denote the distribution radii (defined as the points at which intensity falls to $1/e^2$ of the peak intensity) along the x-axis and y-axis directions, respectively. Next, we only focus on the radius in one direction, denoted as w. This radius can be characterized using a combination of two beam convolutions [4,5]

$$w = \sqrt{w_1^2 + w_2^2} \, , \tag{2}$$

where w_1 and w_2 correspond to the waists of the two beams, respectively. In the case of two identical probes, the corrected beam waist can be determined as $w_1 = w_2 = w_{\min} / \sqrt{2}$, where $w_{\min}$ signifies the spot size measured at the smallest tip-to-tip distances.

References

[1] R. Yan, D. Gargas, and P. Yang, "Nanowire photonics," *Nat. Photonics*, vol. 3, no. 10, pp. 569-576, 2009, https://doi.org/10.1038/nphoton.2009.184.

[2] H. Wu, L. Yang, P. Xu, et al., "Photonic Nanolaser with Extreme Optical Field Confinement," *Phys. Rev. Lett.*, vol. 129, no. 1, p. 013902, 2022, https://doi.org/10.1103/PhysRevLett.129.013902.

[3] L. Yang, Z. K. Zhou, H. Wu, et al., "Generating a sub-nanometer-confined optical field in a nanoslit waveguiding mode," *Adv. Photonics*, vol. 5, no. 4, p. 046003, 2023, https://doi.org/10.1117/1.Ap.5.4.046003.

[4] Y. M. Bian, Y. Li, E. H. Chen, et al., "Free-space to single-mode fiber coupling efficiency with optical system aberration and fiber positioning error under atmospheric turbulence," *J. Opt.*, vol. 24, no. 2, p. 025703, 2022, https://doi.org/10.1088/2040-8986/ac3f8f.

[5] J. B. Decombe, J. F. Bryche, J. F. Motte, et al., "Transmission and reflection characteristics of metal-coated optical fiber tip pairs," *Appl. Opt.*, vol. 52, no. 26, pp. 6620-6625, 2013, https://doi.org/10.1364/AO.52.006620.
